# Effects of intravenous lignocaine, dexamethasone and their combination on propofol-induced pain

**DOI:** 10.6026/973206300212603

**Published:** 2025-08-31

**Authors:** Pragyashri Baghel, Ikraz Patel, Anushree Shukla, Gopal Eekwale

**Affiliations:** 1 Department of Anaesthesia, NSCB Medical College, Jabalpur, Madhya Pradesh, India; 2 Department of Medicine, SAIMS Medical College, Indore, Madhya Pradesh, India; 3 Department of Anesthesia, Apollo Hospitals, Indore, Madhya Pradesh, India; 4 Department of Anaesthesiology, M.G.M. Medical College & MY Hospital, Indore, Madhya Pradesh, India

**Keywords:** Propofol-induced pain, lignocaine, dexamethasone, intravenous anesthesia, pain management, pre-treatment agents

## Abstract

Injection of propofol may provoke pain in up to 70 percent of the patients. In this randomized, double-blind study, the effects of lignocaine, dexamethasone and a combination of both on propofol-induced pain were evaluated in 90 surgical patients. The highest incidence of pain found was in the lignocaine group (70%) and the lowest in the combination group (30%). The combined pretreatment also showed significantly reduced mean pain scores (p < 0.01). The combination of lignocaine-dexamethasone was best effective and this might be embraced in reducing propofol-induced pain.

## Background:

Propofol is the most popular among a large number of intravenously administered anesthesia drugs because of its quick acting, brief acting period and good pharmacokinetics. Although this has its benefits, its disadvantage lies in the pain that a patient is likely to feel during injection as observed with general anesthesia induction where it is estimated that 30 to 70 per cent of the affected patients are likely to feel pain [1]. Although temporary, this pain is capable of affecting patient satisfaction adversely and causing anxiety, particularly where the patient is subjected to his/her first surgery. The propofol-induced injection pain is multifactorial. It is observed that it entails direct inflammation on the venous endothelium and the triggering of the kallikrein-kinin system, which marks the discharge of bradykinin. This enhances violation of vascular permeability and nerve sensitization that boosts the process of nociceptive transmission [2]. Moreover, the size of the vein, the excessively fast rate of injection, and the external temperature of the emulsion are also the factors that enhance the severity of the pain [3]. To reduce this discomfort a number of pharmacological agents are researched opioids, NSAIDs, magnesium sulfate, ketamine, and local anesthetics. Of these lignocaine has proved most reliable. It modulates by eliminating the voltage-gated sodium channels and disrupts the transmission of action potentials and relieves pain at the injection site [4]. Lignocaine used as pretreatment or by coadministration with propofol has revealed great reduction in the incidence and severity of pain as indicated in many studies [5].

As an antiemetic agent, dexamethasone: a synthetic corticosteroid drug that has anti-inflammatory activity as well as a membrane-stabilizing capability is largely utilized in the perioperative period. Recently, it aroused attention in decreasing the pain caused with such intravenous drugs as propofol, possibly by regulating local inflammation and vascular permeability, though the mechanism in this scenario is not quite clear yet [6]. Propofol pain during injection is not merely a factor on physical wellbeing but also a form clinical issue when anesthesiologists aim at providing a smooth induction process. This pain is also something to avoid because it hurts the trust of the patient and reduces perioperative stress [7]. Although lignocaine can be regarded as the usual procedure of pretreatment, dexamethasone has become a possible useful alternative or adjunct agent. Due to their different modus operandi, combination of lignocaine and dexamethasone could provide synergistic effects, since such co-therapy would target both nociceptive and inflammatory mediators of the pain response. Based on theoretical predictions and on early outcomes, it seems that combining two drugs could reduce the discomfort caused by propofol more than each of the drugs separately does [8]. However, limited randomized studies have compared the efficacy of this combination with the individual agents in a clinical setting. This study was designed to evaluate and compare the analgesic effectiveness of intravenous lignocaine, dexamethasone and their combination in reducing propofol injection pain, thereby addressing a gap in the current evidence base [9]. Therefore, it is of interest to evaluate the effects of intravenous lignocaine, dexamethasone and their combination on propofol-induced pain.

## Materials and Methods:

A total of 90 adult patients aged between 18 and 60 years, classified as American Society of Anesthesiologists (ASA) physical status I or II, scheduled for elective surgeries under general anesthesia, were enrolled. Patients with known allergy to propofol, lignocaine, or dexamethasone, those with a history of chronic pain, psychiatric disorders, or on analgesics or corticosteroids, and pregnant or lactating women were excluded.

## Randomization and group allocation:

Participants were randomly allocated into three equal groups (n = 30 each) using a computer-generated random number table and opaque sealed envelopes.

[1] Group A (Lignocaine Group): Received 40 mg of intravenous lignocaine (2 mL of 2%)

[2] Group B (Dexamethasone Group): Received 8 mg of intravenous dexamethasone (2 mL)

[3] Group C (Combination Group): Received 40 mg lignocaine + 8 mg dexamethasone (total 4 mL mixed) intravenously

All pretreatments were administered via a 20G intravenous cannula placed in the dorsum of the hand, 60 seconds before propofol injection.

## Induction procedure:

After standard monitoring (ECG, non-invasive blood pressure, pulse oximetry), baseline vital signs were recorded. Each patient received the assigned pretreatment agent over 10 seconds. One minute later, 2 mL of propofol (1% solution) was injected over 5 seconds, followed by the remaining induction dose titrated according to patient response.

## Pain assessment:

Pain was assessed immediately after the initial 2 mL propofol bolus using a Verbal Rating Scale (VRS) as follows:

[1] 0 = No pain

[2] 1 = Mild pain

[3] 2 = Moderate pain

[4] 3 = Severe pain

The assessment was conducted by an anesthesiologist blinded to group allocation.

## Statistical analysis:

Data were compiled and analyzed using SPSS version 26.0. Categorical variables were expressed as frequencies and percentages, while continuous variables were expressed as mean ± standard deviation. The Chi-square test was used for intergroup comparisons of pain incidence. One-way ANOVA followed by Tukey's post hoc test was applied for comparing mean pain scores among groups. A p-value < 0.05 was considered statistically significant.

## Results:

A total of 90 patients were included and equally distributed across the three study groups (30 patients per group). The groups were comparable in terms of demographic and baseline clinical characteristics. Table 1 (see PDF) shows the demographic distribution among the groups. There were no statistically significant differences in age, gender, or body mass index (BMI) among the groups (p > 0.05), confirming homogeneity of the population. Pain intensity during propofol injection was significantly different across groups. As shown in Table 2 (see PDF), Group C exhibited the lowest incidence and severity of pain, while Group A had the highest frequency of moderate and severe pain. Pain incidence was significantly reduced in Group C compared to Groups A and B (p < 0.001). The mean pain scores were significantly lower in Group C, as displayed in Table 3 (see PDF). Post-hoc analysis confirmed that the combination group had significantly lower scores than either lignocaine or dexamethasone alone (p < 0.05). No major adverse effects were reported in any group. Minor issues like transient flushing or localized discomfort were rare and evenly distributed, as noted in Table 4 (see PDF). Group C (Lignocaine + Dexamethasone) demonstrated the lowest pain scores and highest proportion of pain-free patients. Group A had the highest incidence of moderate to severe pain. The differences among the groups were statistically significant, supporting the superior efficacy of the combination treatment in reducing propofol-induced pain.

## Discussion:

Propofol is a popular drug because its classical properties of a fast onset, short duration of action, and attractive profile characterize the induction of the general anesthetic practice. Nonetheless, one of the greatest pitfalls of its administration is pain caused by intravenous injection hence a major concern to clinicians and the patients. It is indicated that the occurrence of this pain ranges between 30-70 percent, and is thus among the most common adverse side effects caused by the use of propofol drugs as induction agents [2]. Such pain not only impacts patient satisfaction but may impair patient trust in the process of anesthetic care and cause feeling of anxiety and bad thoughts about the surgical process. The mechanism propofol induced pain when injected is complex. It is believed to be direct endothelial venous irritation as a result of the phenol part of the propofol emulsion due to an ensuing activation of the kallikrein-kinin system. This leads to releasing of bradykinin that enhances the vascular permeability and augments transmission in nociceptor [1]. Moreover, the extent of pain felt by the patient is determined by the site of injection, the size of the vein, injection rate and the temperature of the solution given [4]. The above complexities necessitate a reflection on the devising of strategies that are effective enough to reduce this pain without tampering with the pharmacological benefits of propofol. Of the array of agents used in an attempt to deal with this problem, lignocaine has been found to be the most effective in a consistently effective manner. One example of the type of local anesthetic frequently used is lignocaine, which is an amide type of local anesthetic whose mode of action includes inhibition of the voltage-gated sodium channels in the neuron cell membrane, thus preventing the formation and conduction of nerve impulses [6]. As an intravenous injection or as a co-mingled injection, lignocaine has demonstrated effectiveness in predictability when used to decrease the severity and frequency of pain during a needle injection when used before propofol or when propofol and lignocaine together or with other drugs used [5]. The method is easy as well as cheap and it is a widely used method of anesthesia in everyday anesthetic care.

Our results agree with the effectiveness of lignocaine to minimize injection pain; however, what is more important, they also confirm the increased effect of combination of lignocaine with dexamethasone. The potent synthetic corticosteroid, dexamethasone, has proven anti-inflammatory, membrane-stabilizing and endothelial-protective properties. It is traditionally used in the perioperative care to avoid nausea and vomiting, but a potential use in easing injection pain is becoming of interest [8]. Possibly, its capacity to decrease the process of inflammation and prevent leakage of the capillary could assist in alleviating the endothelial irritation evoked by the use of propofol and thus help in perception of less pain [7]. Although dexamethasone singularly led to moderate outcomes in our pain relief trial, a combination of dexamethasone and lignocaine produced better outcomes both with regard to pain incidence and pain severity. The combination therapy reported the fewest average pain scores and the highest number of pain-free patients. This indicates that it may have synergistic effects with lignocaine being a rapid local block treatment and dexamethasone helping to extend and strengthen the effect of the analgesic process via the modulation of inflammatory factors [4]. The results are substantiated by other future researches, as they demonstrated that these dual-mechanism interventions can potentially be more effective than single-agent treatments in the treatment of the propofol-related discomfort [9]. The clinical implication of such findings is immense. An essential component of quality anesthetic care is associated with how comfortable the patient feels during induction. Reduction of procedural pain leads not only to an increase in patient satisfaction but possibly the decrease of additional anxiolytics or sedatives required. Lignocaine and dexamethasone combination have been proven effective, simply practical and relatively safe to use in taking care of this problem. As well as the analgesic effects, dexamethasone has antiemetic coverage contributing to the overall better quality of perioperative stay [10]. Notably, our work has failed to record any adverse effects of serious nature in the use of either of the drugs, either alone or in combination. Mild adverse effects like flushing or temporary local intolerable pain were infrequent and like in all groups, they did not add any safety risk to the combination treatment regimen [11]. This conforms to the safety profiles of the two agents that had been earlier reported at therapeutic doses of use [12]. The monotony of our results with other studies affirms the reliability of our results. Nevertheless, some limitations need to be recognized. The study was carried out in only one location, and the sample size can be regarded as rather small, which can reduce the ability to extrapolate the results to the general population or other clinical environments [13]. Moreover, subjective evaluation of pain scale though it is acceptable, it could be affected by tolerance, level of anxiety and past surgery experiences of a patient [14]. It is suggested that future research with objective biomarkers, more significant sample size and multicentric designs be carried out to interpolate and confirm these studies [15, 16- 17].

## Conclusion:

Intravenous lignocaine and dexamethasone were effective in ameliorating degree and frequency of propofol-related pain more compared to when either of the drugs was used separately. Such synergistic approach is safe, effective and simple to apply in clinical practice. Use of such a mix can help to make patients more comfortable in the event of anesthetic induction.
